# Stabilization of gamma sulfur at room temperature to enable the use of carbonate electrolyte in Li-S batteries

**DOI:** 10.1038/s42004-022-00626-2

**Published:** 2022-02-10

**Authors:** Rahul Pai, Arvinder Singh, Maureen H. Tang, Vibha Kalra

**Affiliations:** Department of Chemical and Biological Engineering, Drexel University, 3141 Chestnut Street, Philadelphia, PA 19104 USA

**Keywords:** Batteries, Electronic devices, Synthesis and processing

## Abstract

This past decade has seen extensive research in lithium-sulfur batteries with exemplary works mitigating the notorious polysulfide shuttling. However, these works utilize ether electrolytes that are highly volatile severely hindering their practicality. Here, we stabilize a rare monoclinic γ-sulfur phase within carbon nanofibers that enables successful operation of Lithium-Sulfur (Li-S) batteries in carbonate electrolyte for 4000 cycles. Carbonates are known to adversely react with the intermediate polysulfides and shut down Li-S batteries in first discharge. Through electrochemical characterization and *post-mortem* spectroscopy/ microscopy studies on cycled cells, we demonstrate an altered redox mechanism in our cells that reversibly converts monoclinic sulfur to Li_2_S without the formation of intermediate polysulfides for the entire range of 4000 cycles. To the best of our knowledge, this is the first study to report the synthesis of stable γ-sulfur and its application in Li-S batteries. We hope that this striking discovery of solid-to-solid reaction will trigger new fundamental and applied research in carbonate electrolyte Li-S batteries.

## Abstract (web summary)

Lithium sulfur batteries are an emerging energy storage medium, but their stability in carbonate electrolyte remains hampered by side-reactions. Here, the authors show that as-produced monoclinic gamma-sulfur on activated carbon nanofibers converts to Li_2_S without the formation of intermediate polysulfides, therefore eliminating irreversible side reactions and improving cycling stability.

## Introduction

State of the art lithium–sulfur (Li–S) batteries are attractive candidates for use in electric vehicles (EVs) and advanced portable electronic devices owing to an order of magnitude higher theoretical energy density than the conventional lithium-ion batteries (LIB)^1–3^. In addition, sulfur is both environmentally friendly and naturally abundant in the earth’s crust. However, the current Li-S system is plagued by numerous challenges^4,5^. The insulating nature of both sulfur and the final discharge product, Li_2_S, results in low material utilization during the redox processes. A bigger challenge is the dissolution of the intermediate reaction products, lithium-polysulfides (LiPs), into the electrolyte causing the well-known “shuttle-effect”^4^. Polysulfide shuttle results in an uncontrollable deposition of sulfide species on the lithium metal anode reducing coulombic efficiency and increasing capacity fade^6^. This series of challenges have been extensively studied in the past decade with most studies being in the ether electrolyte-based Li–S batteries^7–12^. A much less discussed, but debilitating drawback for the commercial viability of Li–S batteries is the use of the ether electrolyte itself. Ether-based solvents are highly volatile and have low flash points posing a significant risk of operating such batteries beyond room temperatures^13–15^ For example, dimethoxyethane (DME), an important ingredient used in present-day Li–S batteries has a boiling point of only 42 °C^16^. Therefore, despite tremendous research in overcoming Li–S battery challenges, the practicality of such battery chemistries is severely hindered due to severe safety concerns and transport issues^17^.

LIB have been dominant in the commercial market for the past 30 years with the use of carbonate-based electrolytes, well known for their reasonably safe behavior beyond room temperature (typical boiling points of >200 °C) and wide operational window^18–20^. In addition, flame retardant additives have been extensively researched, designed, and applied for carbonate-based electrolytes to enhance their reliability^20^. Hence, the tremendous knowledge gained on carbonate electrolytes in the Li-ion battery field over the past three decades can potentially be applied for the future development of Li–S batteries. However, it is known that when carbonate electrolyte is used in Li–S batteries, an irreversible reaction between carbonate species and polysulfides takes place to form thiocarbonate and ethylene glycol, terminating further redox reactions and shutting down the battery^21^. A handful of reports have recently demonstrated the use of Li–S batteries with carbonate-based electrolytes with stable and reversible capacity^22–27^. These papers propose a few different concepts/hypotheses that potentially enable successful battery operation in carbonate electrolytes. A common feature in these works is the nano-confinement of sulfur. For example, Xin et al. synthesized sulfur cathodes via confining sulfur molecules into 0.5 nm pores of microporous carbon host materials^25^. They proposed that the confinement within sub-nano pores prevented the formation of larger sulfur allotropes (S_5–8_) and possibly resulted in small sulfur allotropes (S_2–4_) only, which in turn converted to Li_2_S without the intermediate polysulfides (Li_2_S_8_, Li_2_S_6_…). They showed stable capacity (with single discharge plateau) in carbonate electrolytes for up to 200 cycles. However, it is not clear how the smaller allotropes exhibited a capacity close to the theoretical capacity of S_8_→Li_2_S conversion. In another work, Fu *et al*. also synthesized carbon/sulfur cathodes with sulfur confined in sub-nanometer carbon pores (0.4–1 nm)^26^. Their material also exhibited single plateau discharge and stable reversible capacity for 100 cycles in carbonate electrolyte. They proposed that the small pore size forced the de-solvation of lithium ions and resulted in solid-state lithiation and de-lithiation of confined S_8_ molecules. Overall, these works propose stringent pore size requirements (<0.5 nm) for the host carbon requiring complex synthesis procedures limiting broad deployment, while also theoretically limiting the possible sulfur loading (due to limited available volume of precisely sized micropores). Moreover, none of these reports attempt to characterize the initial sulfur allotropes (reactants) nor the discharge or charge products formed, and therefore the source of energy storage/capacity is unclear. Moreover, to the best of our knowledge, there are no reports employing sulfur via a non-confinement approach in carbonate-based electrolytes in the Li–S system.

In this study, we synthesize and study a novel phase of sulfur (γ-monoclinic phase) in carbonate-based Li–S batteries. We demonstrate that despite an exposed “un-confined” deposition of this sulfur phase on the host carbon material, the carbonate-based battery exhibits high reversible capacity, which stabilizes to 800 mAh·g^−1^ in the first few cycles and then it remains stable with a small 0.0375% decay rate over 4000 cycles. The cells exhibit a high capacity of 650 mAh·g^−1^ even after the end of 4000 cycles. The host electrode consists of freestanding, binder, and current collector-free carbon nanofibers (CNFs). After sulfur deposition and slow cooling at room temperature in an autoclave developed in-house, sulfur adopts the rare monoclinic γ-phase rather than the typical orthorhombic α-phase on the surface of CNFs. This phase remains stable at room temperature for over a year with no apparent evidence for phase change even beyond this timeframe. Electrochemical characterization and post-mortem spectroscopy/microscopy studies on cycled cells reveal an altered redox mechanism that reversibly converts monoclinic sulfur to Li_2_S without the formation of intermediate polysulfides for the entire range of 4000 cycles. The development of unconfined high loading sulfur cathodes in Li–S batteries employing carbonate-based electrolytes can revolutionize the field of high energy density practical batteries.

## Results and discussion

### Material characterization

**Fig. 1:**
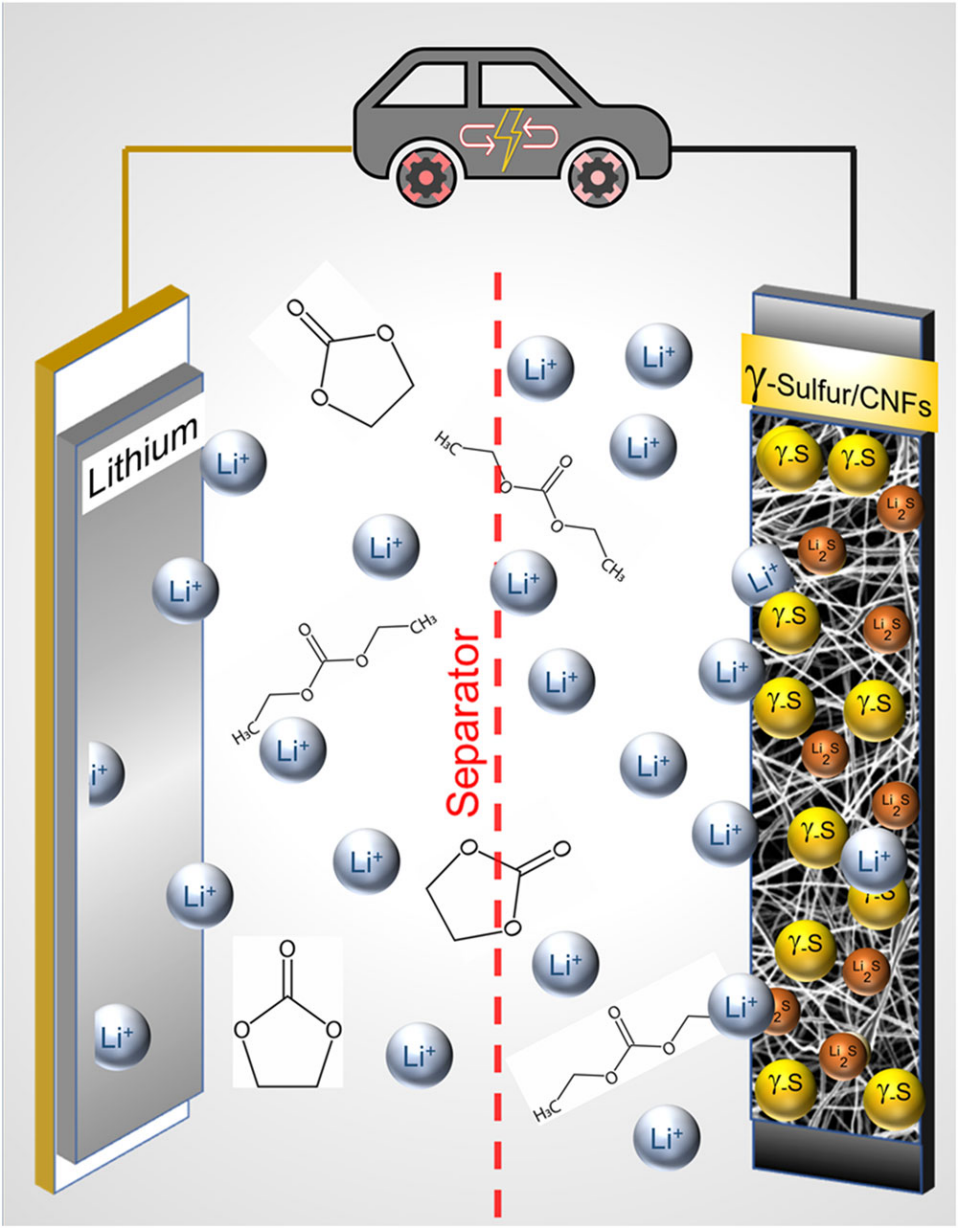
Schematic of a lithium-sulfur battery discharge in the carbonate-based electrolyte. The gamma-monoclinic sulfur is deposited on the external surface of the carbon nanofibers. The yellow balls signify surface deposited gamma-monoclinic sulfur, red balls signify lithium sulfide, the product formed after the reduction of sulfur.

**Fig. 2:**
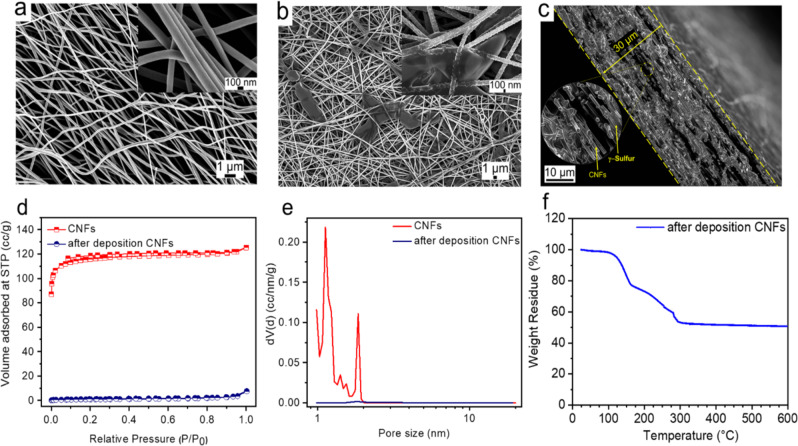
Material characterization of CNFs. **a** SEM images of CNFs before deposition. **b** SEM images of CNFs after sulfur deposition, (inset) zoomed-in image of well-deposited sulfur particles of CNFs. **c** Cross-sectional SEM image of CNFs after deposition showing sulfur deposition throughout the cathode. **d** Isotherms obtained before and after sulfur deposition on CNFs. **e** The pore size distribution of CNFs before and after sulfur deposition. **f** TGA curves of sulfur deposited CNFs in an argon environment.

Figure 1 provides a schematic outline of a Li–S cell with the monoclinic gamma-sulfur-based cathode in carbonate electrolyte. The scanning electron microscopy (SEM) images in Fig. 2a show a smooth CNF surface with an average diameter of ~150 nm. After sulfur deposition in the autoclave, SEM images reveal a consistently rough fiber morphology suggesting a uniform and conformal coating of sulfur (Fig. 2b). Few regions display blocks of sulfur deposited within the inter-fiber spacing. Overall these images provide clear evidence that the sulfur is largely on the outer CNF surface. To further understand the effect of sulfur deposition on surface area and pore sizes of CNFs, a BET surface area analysis was conducted. Figure 2d, e shows the N_2_ absorption/desorption isotherm plots and pore size distribution of CNFs before and after sulfur deposition. For CNFs, the gas uptake increases to a high value at low relative pressure (*P*/*P*_0_ < 0.05), and the adsorption isotherm exhibits a plateau at middle and high relative pressures. The hysteresis loop at *P*/*P*_0_ = 0.2–1.0 represents mesoporosity. The adsorption isotherm is a combination of IUPAC types I and IV isotherms which confirms the presence of both micro and mesopores on CNFs^23^. However, after the sulfur deposition in the autoclave, the isotherm shows significantly lower gas uptake suggesting a decrease in surface pores. Pore size distribution in Fig. 2e shows that the CNFs portray a multi-modal pore structure in the nanoscale regime. After the sulfur deposition, the CNFs display an enormous reduction in surface area (3.14 m^2^ g^−1^) suggesting pore filling by sulfur. Pore structural parameters of all the materials are summarized in Table 1. The BET and SEM data suggest that sulfur is partially confined within the carbon nanopores. Nevertheless, there is clear evidence of exposed unconfined sulfur on the external carbon surface. Figure 2f shows the thermogravimetric analysis on sulfur-deposited CNFs conducted in an inert nitrogen atmosphere with a heating rate of 10 °C· min^−1^ from room temperature to 600 °C. The thermogravimetric analysis (TGA) curve shows mild initial weight loss below 100 °C associated with evaporation of adsorbed moisture. Beyond 100 °C, we observe a continuous weight loss over a wide temperature window until 300 °C with two distinct degradation rates. The melting of sulfur occurs at 119 °C and sulfur starts to evaporate. The wide decomposition temperature range and multiple degradation rates observed in TGA further corroborate partial pore-confinement of sulfur. While the higher-rate lower-temperature weight loss suggests evaporation of exposed unconfined sulfur, the lower-rate higher-temperature loss can be attributed to sulfur confined in micro/mesopores. The sulfur content in the CNFs/S composite determined by TGA was ~50 wt%.

**Table 1:** Surface area and pore volume measurements of CNFs before and after thermal treatment.

Sample	S_BET_ (m^2^ g^−1^)	Average pore diameter (nm)
CNFs before thermal treatment	458	1.12
CNFs after thermal treatment	3.14	n/a

**Fig. 3:**
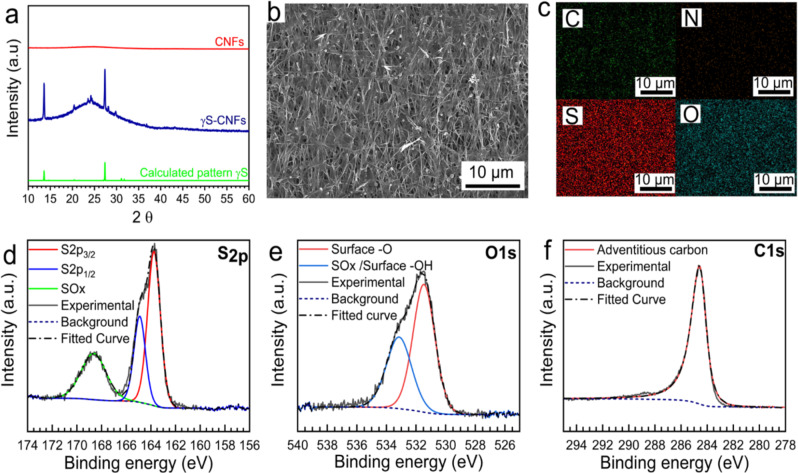
Phase and surface characterization of CNFs and γ-CNFs. **a** XRD pattern of CNFs, γS-CNFs, and calculated pattern of γS. **b**, **c** SEM image of γS-CNFs showing well-distributed sulfur deposition and EDS elemental mapping. **d**–**f** XPS spectra of S, O, and C on the γS-CNFs.

Figure 3a shows the room temperature X-ray diffraction (XRD) patterns of bare CNFs and after sulfur deposition. The bare CNFs display no significant diffraction peaks. A wide hump is seen around 2 theta of 20°–30° due to the amorphous nature of carbon in CNFs. However, after the sulfur deposition, we see a rare and metastable phase of sulfur—the monoclinic γ phase. This is striking behavior as out of 24 sulfur allotropes discovered in the last 200 years, only orthorhombic-alpha (S_8_), rhombohedral (S_6_), hexagonal (S_8_), and polymeric allotropes are known to be stable at room temperature^28,29^. Of these, orthorhombic alpha is the most stable phase of sulfur at room temperature. The only well-understood monoclinic phase is the β-monoclinic that is known to appear on heating α-orthorhombic to >94.4 °C. Below this temperature, the β-monoclinic phase quickly converts back to the stable α-orthorhombic^28–30^. Nevertheless, repeatable XRD signatures after sulfur deposition treatment show the presence of γ-monoclinic sulfur in our samples at room temperature. Out of the various cyclo-octa sulfur polymorphs, γ-monoclinic sulfur is rarely observed and is known to be found in oil wells and has a melting point of 106.8 °C^28^. Nevertheless, little to no progress has been made towards the development and understanding of this structure^30^. Only a handful of reports (<5) in the past two centuries have even mentioned the presence of γ-monoclinic sulfur at room temperature with a short lifetime^31,32^. For example, Watanabe in 1974 confirmed the synthesis of γ-monoclinic sulfur (γ-S) by treating cuprous ethyl xanthate with pyridine, however, the γ-S crystals soon converted to the stable orthorhombic α sulfur at room temperature^30^. In our case, we have studied the stability of our (γ-S) phase for 2+ years at room temperature and it remains stable, showing no signatures of the phase change (Supplementary Fig. 1).

However, why sulfur stabilized with a monoclinic gamma crystal structure after deposition in our samples is currently unclear. A recent DFT study on the stabilization of metastable sulfur shows that the carbon host can facilitate the stabilization of a monoclinic sulfur phase if the number of carbon atoms exceeds 0.3 per S_8_ unit crystal structure^33^. In addition, it was recently suggested by Moon et al. that carbon facilitates the formation and helps in retaining the monoclinic structure at room temperature for longer periods^31^. In our study, we hypothesize that the (γ-S) phase formed at elevated temperatures penetrates the porous carbon structures and retains its crystal structure even after cooling due to the local carbon density within the pores. This unique crystal structure once trapped within the pores possibly propagates throughout the sulfur blocks including those that are externally deposited in an “unconfined” state.

Figure 3b, c shows the EDX mapping and corresponding low magnification SEM image exhibiting the uniform distribution of sulfur. To confirm the chemical composition and surface properties of pristine γ-monoclinic-sulfur-based CNF cathodes (γS-CNFs), XPS measurements were performed and the results are displayed in Fig. 3d–f. The survey spectra (Supplementary Fig. 2) show the existence of C_1s_, S_2p_, and O_1s_ peaks in the composite. The peaks centered at 284.6, 531.0, and 533 eV spectra correspond to the C1s, O1s, and the adsorbed surface hydroxyl group (−OH), respectively^34^. Figure 3d displays the high-resolution S_2p_ spectra of the composite. The S 2p_3/2_ peak at 163.7 eV and S 2p_1/2_ peak at 164.9 eV with an area ratio of 1:2 and Δ*E* of 1.18 eV are the characteristics of solid sulfur in the composite^35^. Another broad peak centered at 168.8 eV can be attributed to the surface oxidation of sulfur during high-temperature sulfur deposition treatment. The smooth Lorentzian asymmetric peak of carbon further confirms that sulfur does not react with the bare carbon surface.

### Electrochemical characterization

**Fig. 4:**
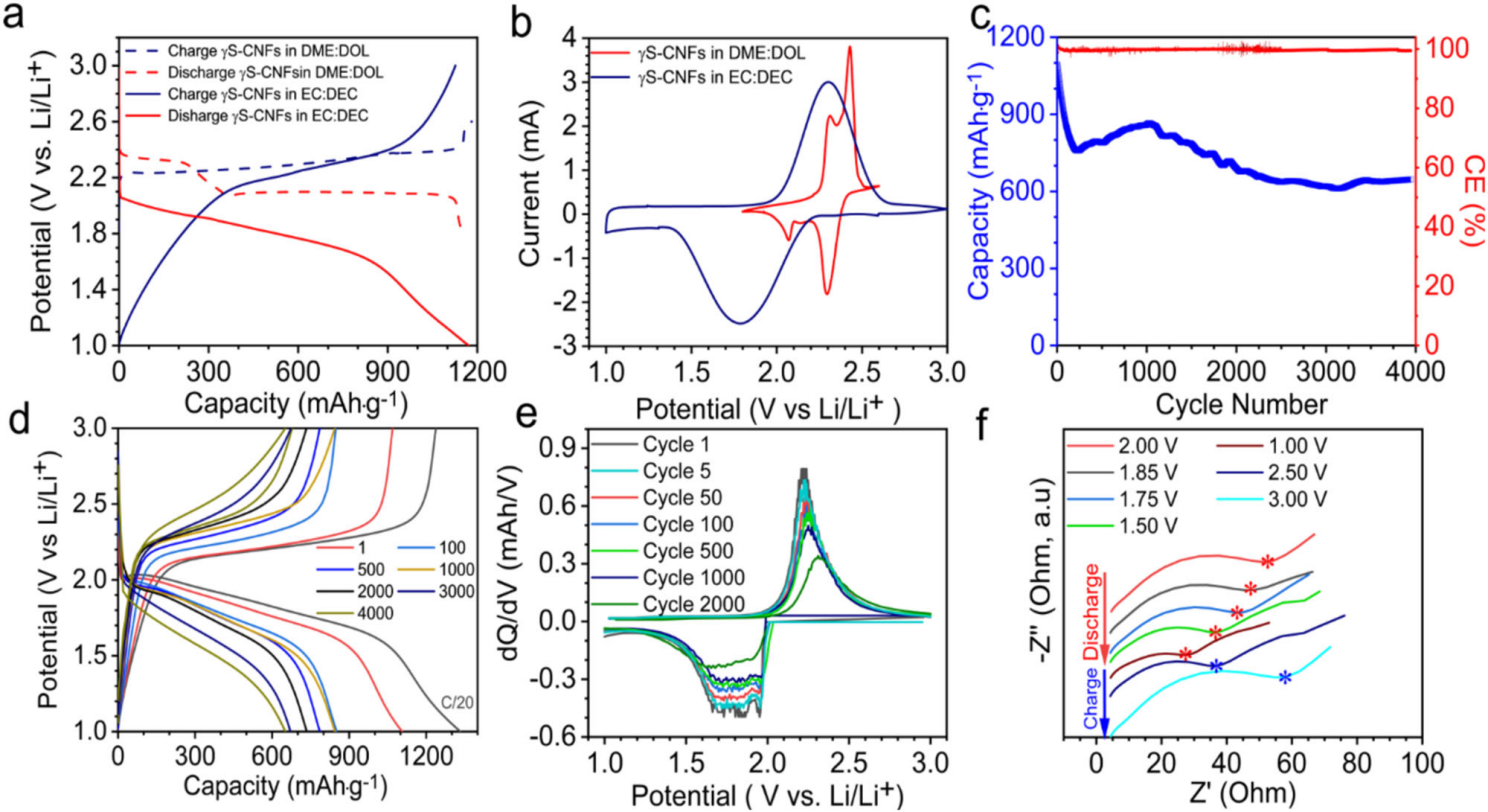
Electrochemical characterization γS-CNFs. **a** Charge–discharge patterns of γS-CNFs in ether electrolyte (DME: DOL) and carbonate electrolyte (EC: DEC). **b** Cyclic voltammetry curves of γS-CNFs in ether electrolyte (DME: DOL) and carbonate electrolyte (EC: DEC) at 0.1 mV·s^−1^. **c** Cycling stability of γS-CNFs in EC: DEC at a current rate of 0.5 C. **d** The charge–discharge profiles of γS-CNFs at various cycle numbers. **e** Differential capacity analysis plot of γS-CNFs displaying a single peak in the charge–discharge cycle. **f** Nyquist plot of γS-CNFs cathode as a function of voltage during the charge–discharge cycle. The cells were assembled with S loading of 0.5 mg·cm^−^^2^ using ~50 wt% sulfur in the cathode and an E/S ratio of 20.

Figure 4 shows the electrochemical performance evaluation of γS-CNFs used as free-standing cathodes in CR 2032 type coin cells with reference/counter as lithium. Both ether and carbonate-based electrolytes were employed to understand the electrochemical phenomenon in each system. Due to the insulating nature of sulfur, electrochemical impedance spectroscopy (EIS) was first performed on the sulfur cathode with and without resting time (Supplementary Fig. 3). Initially, the results demonstrate a small surface charge transfer resistance (~110 ohm) indicating good conductivity of the electrode and efficient interfacial contact which further decreases with resting time (~30 ohm). The electrode demonstrates a reversible electrochemical redox behavior in both electrolytes. However, the charge-discharge profiles are drastically different (Fig. 4a). The ether electrolyte charge-discharge profile exhibits a standard two-plateau behavior as reported in most prior literature reports^1,6^. The first plateau at 2.3 V is attributed to the conversion of sulfur to long-chain polysulfides and the second plateau at 2.1 V represents the conversion of long-chain polysulfides to Li_2_S_2_ and Li_2_S (2.1 V). However, the same γS-CNFs cathodes in carbonate electrolytes demonstrate a single plateau at 2.0 V in the first and all consecutive cycles during discharge and 2.2 V in charge profiles suggesting the possibility of a polysulfide digression route to directly form lithium sulfide in carbonate electrolyte. This solid-to-solid conversion possibly also leads to a higher overpotential explaining the lower plateau voltage observed in carbonate electrolytes. The electrochemical behavior is consistent with CV profiles, wherein the cells with ether electrolyte show two peaks, while the cells with carbonate electrolyte only show a single peak as shown in Fig. 4b.

Figure 4c shows the long-term cycling data for cells made using carbonate electrolyte tested at 0.5 C current rate. The cells exhibit a capacity of 800 mAh·g^−1^ after the first few cycles with only 0.04 % decay beyond that. The cells still retain a capacity of 658 mAh·g^−1^ even after 4000 charge-discharge cycles. To the best of our knowledge, this is the highest achieved reversible capacity after 4000 cycles to date. The initial drop in capacity may be attributed to the loss of contact due to volume expansion during cycling which stabilizes as the cycling proceeds. It is well-known in the literature that pure uncoated lithium anodes are unstable and commonly have poor rate capabilities since the high current would dramatically promote the formation of lithium dendrites. Nevertheless, to keep the focus of our work on the development of the cathode and its ability to perform with single plateau behavior in carbonate electrolyte, we used excess (thick) lithium, so the anode is not expected to be a limiting factor. *Postmortem* digital images (Supplementary Fig. 4) after opening the cell show a thick layer of dead porous lithium on the anode. Scraping the top part shows a clear lithium anode. We believe the thick lithium foil used as counter/reference electrode in our cells contributes towards long-term cycling. However, in the future, anode stabilization strategies are required to reduce the thickness of Li, minimize continuous side reactions of Li with electrolyte, and for uniform Li deposition As shown in Fig. 4d, the discharge profile continues to exhibit a single plateau through the entire cycle life of 4000 cycles. The reduction in capacity after cycling is possibly due to the reaction of carbonate electrolytes. It is established from a recent report by Yim et al. that polysulfides (if generated) attack carbonate species via nucleophilic substitution reaction to form irreversible products—thiocarbonate and ethylene glycol—and shut down further electrochemical activity after the first cycle^21^. Therefore, our data suggest that these γS-CNF-based cells continue to follow a polysulfide digression route through the entire cycling in carbonate electrolyte, which explains not only continued battery operation despite the presence of carbonate species but also our excellent cycle stability of 4000 cycles. For comparison of long-term stability, we have shown cycling data of γS-CNF in ether and carbonate electrolyte with similar sulfur loading and the current rate of 1C (Supplementary Fig. 5). The cathode in ether electrolyte follows a standard route with polysulfides as the intermediate products with two-plateau discharge. Here we see a gradual decline in capacity due to the expected polysulfide shuttling and subsequent loss of active material. To demonstrate the detrimental effects of shuttling in ether electrolyte we performed the cycling test with and without the addition of LiNO_3_ since it plays a key role in passivation of lithium surface (Supplementary Fig. 6). The results show improved stability and gradual capacity fade by the addition of LiNO_3_.

To further corroborate this unique electrochemical behavior of γS-CNFs in carbonate electrolytes and infer information about the reaction mechanism, we conducted differential capacity (d*Q*/d*V*) analysis and EIS as a function of voltage. We see a consistent single peak in the d*Q*/d*V* plot for 2000 cycles further strengthening our finding of a single-phase conversion. The peaks minimally shift during cycling suggesting good material integrity and minimal increase in resistance during cycling. As a next step, the EIS measurements of the lithium half-cells with γS-CNFs as composite cathodes were carried out at various potentials during charge–discharge cycles. Although owing to the complexity of any battery assembly, a straightforward and quantitative interpretation of the EIS data is non-trivial. Nevertheless, it can provide powerful information on qualitative trends. Figure 4f presents the typical Nyquist plots for our Li–S batteries illustrating their impedance trends as a function of voltage. As seen in this figure, a typical Nyquist plot consists of a semicircle in the high frequency to medium frequency range, which is attributed to the interfacial charge transfer resistance. The charge transfer resistance (*R*_ct_) and series resistance monotonically decrease as the cathodic curve progresses towards a lower potential for the entire discharge cycle. The trend is reversed when the battery is charged back to a higher potential. This observation contrasts with the literature, wherein the *R*_series_ first decreases and then increases back again in the same discharge cycle due to the formation of soluble polysulfides at intermediate voltages^36–38^. These intermediate polysulfides significantly lower the *R*_s_ and *R*_ct_ due to the disappearance of both of the solid insulating materials—the initial reactant, sulfur, and final product, Li_2_S. It is worth noting that in the literature, *R*_ct_ of the final discharged cell still remains lower than the initial *R*_ct_ (at OCV) due to the reduced resistance of Li_2_S compared to pure sulfur^39^. A monotonic decrease in *R*_s_ and *R*_ct_ during discharge in our work provides further evidence that we are eliminating the formation of polysulfides.

**Fig. 5:**
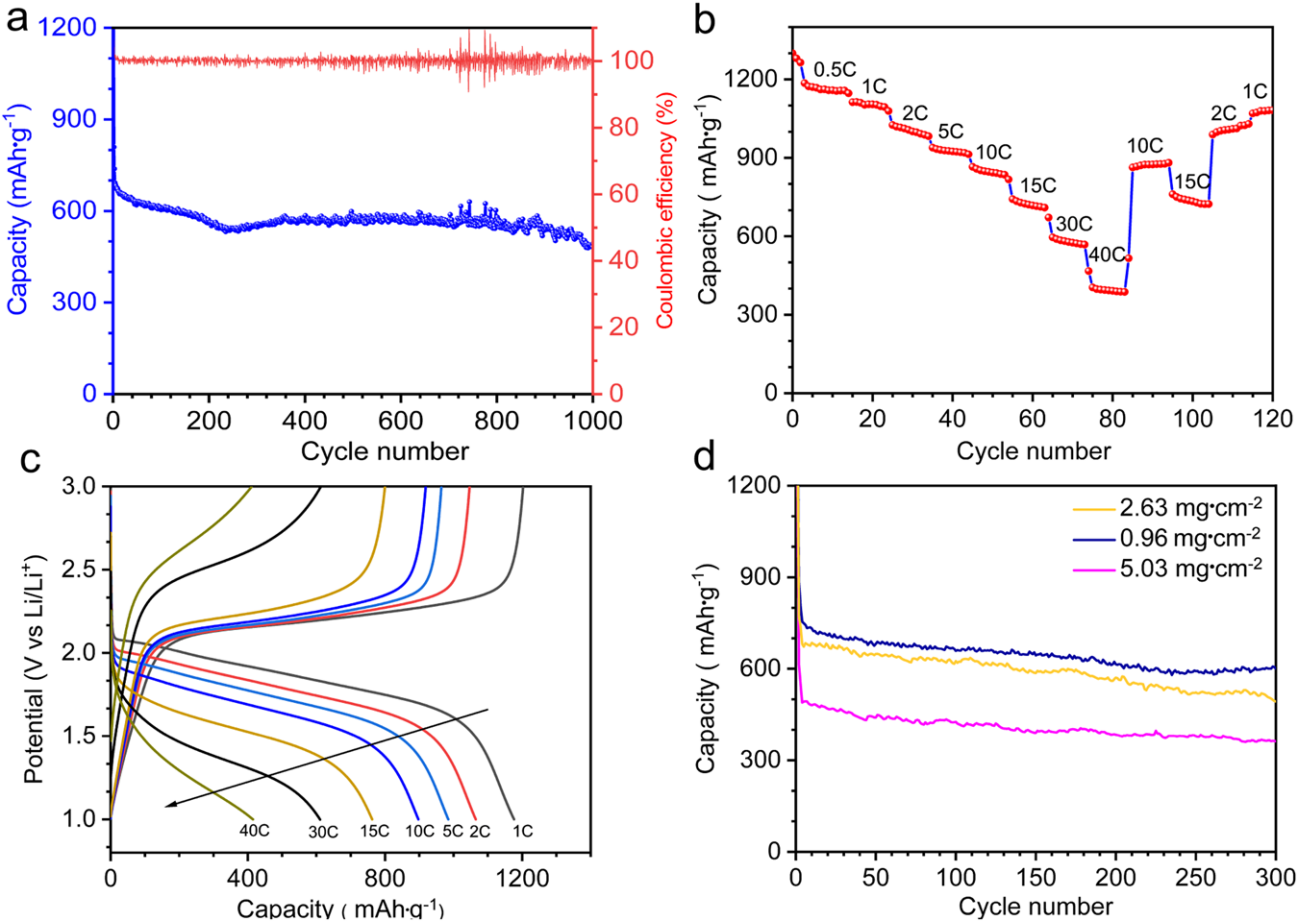
Rate performance and high loading analysis of γS-CNFs. **a** Long cycling of γS-CNFs at a low current rate 0.1 C at 1.2 mg·cm^−2^ loading. **b** Rate performance of γS-CNFs in carbonate electrolyte at 0.5 mg·cm^−2^. **c** Charge–discharge profiles of γS-CNFs at various C rates. **d** Long cycling as a function of higher loading. The cells were assembled with variable S loading of 1.2–5.03 mg·cm^−2^ using ~50 wt% sulfur and an E/S ratio of 20.

To evaluate the practical application of our carbonate-based Li–S system, we cycled our cells with γS-CNFs cathodes at various C rates and loadings. As shown earlier, these batteries demonstrate stable capacity at 0.5 C rate for over 4000 cycles. To demonstrate battery operation at harsh conditions, we tested the batteries for long-term cycling at 0.1 C (Fig. 5a). The batteries provided stable ~550 mAh·g^−1^ capacity for over 1000 cycles with a small 0.0015% decay and coulombic efficiency ≧99%. In addition, these batteries show excellent rate performance with a capacity of 1170, 1080, 980, 900, 750, 600, and 410 mAh·g^−1^ at 1, 2, 5, 10, 15, 30, and 40 C, respectively (Fig. 5b). It is interesting to see these cells exhibiting a capacity of 400 mAh/g even at 40 C corresponding to discharge and charge time of only ~30 s. The traditional ether-based batteries perform only up to 2 C at which the performance deteriorates significantly. Figure 5c shows that our cells exhibit a similar single plateau discharge at all C rates. Such rate capability suggests efficient nanoscale contact between γ-monoclinic sulfur and the host CNFs and good interfacial electrode–electrolyte contact owing to the 3D inter-fiber porous architecture. Furthermore, the binder-free freestanding format of the CNF host, we believe, provides uninterrupted electron pathways despite the presence of insulating sulfur. This is unique compared to traditional slurry-based cathodes where carbon and sulfur powders are mixed together with limited to no control over spatial morphology deteriorating overall composite conductivity. Figure 5d shows the cycling data for higher commercially relevant sulfur loadings. Cells with 5 mg·cm^−^^2^ of sulfur demonstrate stable cycling for 300 cycles at 0.1 C (2.35 mAh·cm^−2^). This finding demonstrates that unconfined sulfur deposition using γ-S-CNF can pave the path toward commercially relevant sulfur loadings in carbonate electrolytes.

Exposed unconfined sulfur in the past has been associated with irreversible reactions with carbonate electrolyte and battery shut down after the first cycle as shown by Kim et al. On comparing our work with these previous unsuccessful sulfur studies in carbonate electrolytes, the striking difference is the crystal structure of the sulfur in our cathodes. Most Li–S literature, regardless of the electrolyte, uses α-orthorhombic sulfur, which is the most stable sulfur allotrope at room temperature. It is therefore likely that the single plateau behavior seen reversibly and consistently for 4000 cycles in our work is directly linked to the role of γ-monoclinic phase.

Recently, Kaskel and coworkers demonstrated the effect on cathode electrolyte interphase (CEI) utilization of porous carbon structures in Li-S batteries for their use in carbonate-based electrolytes^27^. They utilized commercial micro/mesoporous carbon as well as in-house developed carbide-derived microporous carbon. They have shown the development of SEI on the cathode (CEI) results in a high irreversible capacity loss in the first cycle, wherein the discharge capacities obtained are beyond the theoretical capacity of sulfur. This increase in capacity in the first cycle is attributed to the decomposition of the organic electrolyte as the potential is swept below 1 V wrt Li/Li^+^ for the formation of CEI in the first cycle. In addition, Aurbach et al., have demonstrated decomposition of ionic liquids (ILs) and organic electrolytes for the development of CEI on cathode interphase and concluded that the formation of SEI plays a dominant role in enabling sulfur utilization and not the confinement of sulfur in pores^40^. However, in our case, we see stable performance in cyclic voltammetry and charge–discharge cycles without subjecting our cathode below 1 V in the organic electrolyte from the initial cycling period. Although we observe the loss of capacity during cycling, the cathodes never achieved capacities beyond theoretical values. This striking difference in electrochemical response differentiates our behavior compared to CEI-based work demonstrated in the literature.

To understand the importance of CNF based substrate for the deposition of γ-monoclinic sulfur contributing towards its stability we utilized a commercial microporous/mesoporous carbon substrate (C Novel, MH-00, Toyo Tanso, Japan). Upon the use of the same thermal treatments utilized for deposition of γ-sulfur, XRD results reveal a broad amorphous peak corresponding to carbon with no signature of sulfur. In addition, electrochemical results shown in Supplementary Fig. 7 demonstrate an extremely low capacity (1–3 V range) with triangular charge-discharge profiles, further suggesting the importance of CNFs for γ-monoclinic sulfur deposition and its subsequent utilization in Li–S batteries.

Furthermore, to state the importance of monoclinic crystal structure on performance, we utilized the scraped residual material deposited on the top wall of our autoclave for cathode fabrication. The brownish shiny material has an XRD pattern showing mixed phase with few peaks corresponding to γ- monoclinic sulfur (Supplementary Fig. 8). Nevertheless, it is worth noting that this mixed-phase cathode yielded a single plateau charge-discharge similar to pure γS-CNFs in carbonate electrolyte and functions stably in carbonate electrolyte for close to 500 cycles (Supplementary Fig. 8c). The specific capacity is relatively lower possibly due to the absence of pure gamma phase resulting in lower sulfur utilization. Nevertheless, we still achieve 400 mAh/g capacity in carbonate electrolyte despite a non-activated carbon substrate confirming the role of gamma sulfur in the observed electrochemical behavior.

A possible reason for such a staggering effect of sulfur crystal structure could be the difference in phase density. While there are discrepancies in the reports on densities of various sulfur allotropes as synthesizing a metastable allotrope is non-trivial, Meyer et al. did groundbreaking work on sulfur allotropes in the early 1960s. He reported a density of γ-S to be higher than its α-counterpart (2.19 g·cm^−3^ vs. 2.069 g·cm^−3^)^28,29^. The close compactness within the γ-monoclinic crystal structure possibly provides greater stability and easy lithiation into gamma monoclinic crystal structure in the carbonate electrolyte. In the ether electrolyte, we believe that γ-sulfur converts to a more favorable phase to yield a two-plateau discharge. A study on the stability of this unique sulfur crystal structure in various electrolytes is underway.

While providing experimental evidence for why the γ-monoclinic phase alters the discharge mechanism is non-trivial and will require future computational studies, we conducted post mortem studies using XRD and XPS to understand the redox products after charge and discharge cycles and to provide evidence that the stable capacity is indeed largely a result of the desired sulfur to Li_2_S reactions (and not any unwanted degradation reactions). This is also particularly important as most papers reporting single plateau discharge profile in Li–S batteries do not provide reactant and/or product characterization for a deeper understanding of the charge storage mechanism and to evaluate electrolyte decomposition (if any). Below we discuss both *post mortem* spectroscopy and microscopy data.

### Postmortem SEM and TEM analysis

**Fig. 6:**
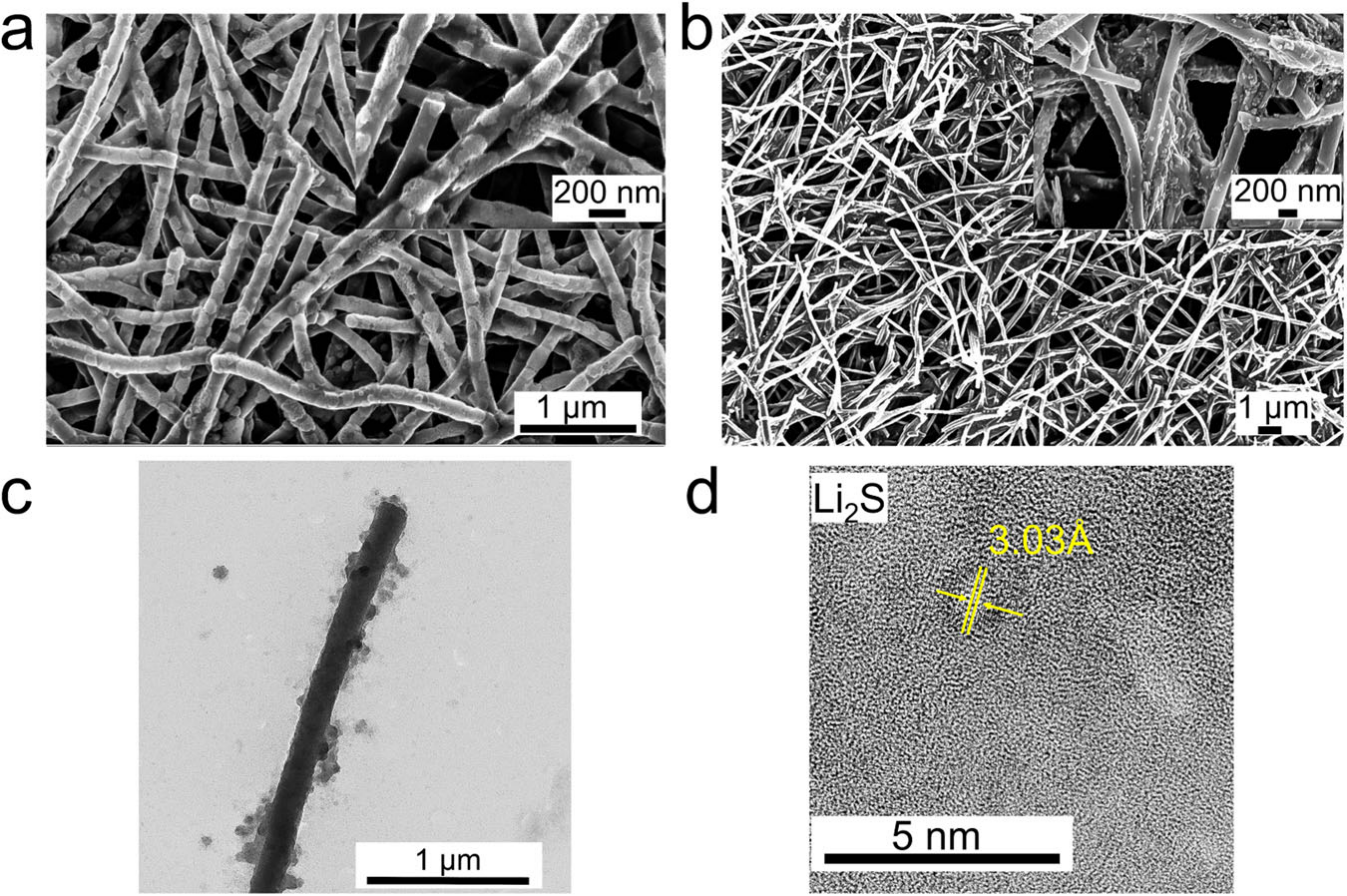
Post-mortem SEM and TEM analysis of γS-CNFs after charge–discharge cycles. **a** SEM images of γS-CNFs after 20 discharge cycles. **b** SEM images of γS-CNFs after 20 charge cycles. **c** TEM image after 1000 cycles. **d** HRTEM image after discharge cycle.

To understand chemistry and surface morphology after cycling, we conducted postmortem microscopy of cycled cells. The surface morphology γS-CNFs after 20 charge and discharge cycles at 0.05 C is shown in Fig. 6a, b. Compared to pristine samples, the charged and discharged samples still retain their freestanding architecture. However, the surface deposited γ-sulfur redistributes itself on the surface possibly due to volume expansion–contraction during discharge–charge cycles. Nevertheless, γ sulfur still remains exposed and unconfined on the surface of CNFs.

Figure 6c shows a TEM image taken after lithiation in a completely discharged sample post-1000 cycles. Despite ultrasonication for TEM sample preparation, the sulfur particles appear to be well-adhered to the CNFs. An HRTEM image (Fig. 6d) taken from the deposited structures of discharged γS-CNFs confirms the formation of Li_2_S as its lattice fringe width was found to be 3.30 Å corresponding to the (111) orientation of the Li_2_S cubic phase.

### Postmortem XPS and XRD analysis

**Fig. 7:**
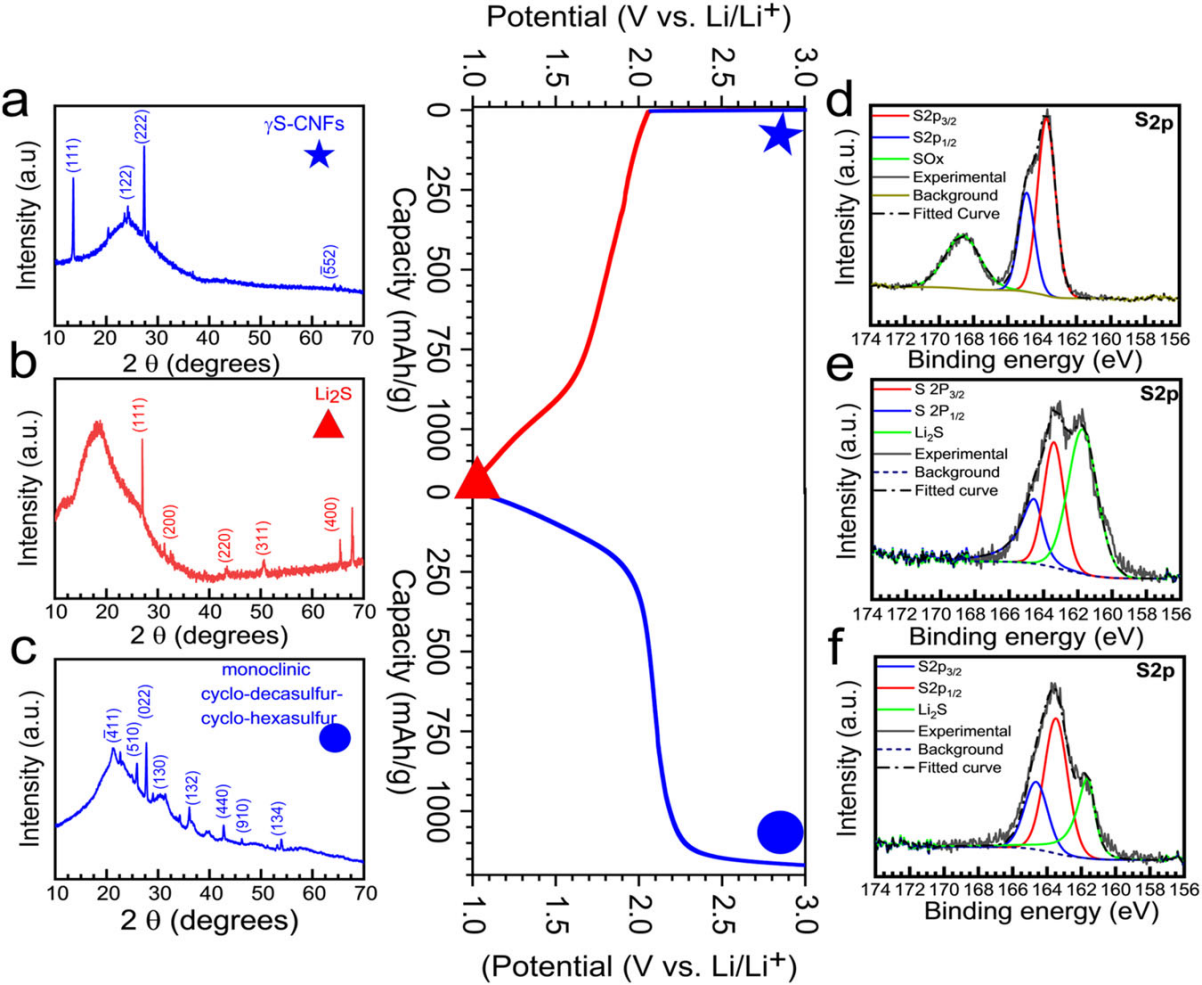
Post-mortem XRD and XPS analysis of γ-S/CNFs after charge–discharge cycles. **a**–**c** XRD pattern of a. pristine γS-CNFs. **b** Discharged γS-CNFs showing conversion to Li_2_S. **c** Charged XRD pattern showing conversion to monoclinic cyclo-hexa-cyclo-deca sulfur. **d**–**f** High-resolution S2p XPS spectra of (**d**). Pristine γS-CNFs. **e** Discharged γS-CNFs displaying Li_2_S peak. **f** Charged γS-CNFs showing the presence of sulfur peak. **g**–**f** High-resolution F1s spectra of g discharged γS-CNFs, f charged γS-CNFs.

Figure 7 provides the postmortem XPS and XRD data both after discharge and after charge. In the center of the figure, we show a typical charge-discharge voltage profile that we obtain for our samples and the specific points where spectroscopy data was collected after fifth discharge and after fifth charge cycles at 0.05 C. Prior to XPS analysis, the cycled samples were thoroughly rinsed with the EC:DEC solvent and let to dry out under Ar atmosphere and later under dynamic vacuum for 48 h. The samples were then loaded in an XPS transfer assembly in the glove box and transferred to the XPS vacuum chamber avoiding any contact with the ambient atmosphere.

As discussed earlier, in the pristine sample with vapor-deposited γS-CNFs, we see the presence of adventitious carbon, C at 284.6 eV from the CNF surface. The S 2p spectra show the presence of sulfur doublet peaks (S 2p_3/2_ and S 2p_1/2_) positioned at 163.7 and 164.9 eV with a peak separation of 1.18 eV. In addition, we see a peak at higher binding energy (168.94 eV) associated with the formation of surface oxides (S–O) during high-temperature deposition. A similar bond can be seen in O1s spectra, wherein the peak at 531.86 eV is attributed to the surface oxides. After complete discharge, the S_2p_ spectra show the appearance of a new strong peak at a lower binding energy of 161.8 eV associated with Lithium sulfide (Li_2_S) deposition. Interestingly, we also note the presence of a new peak at 685.5 eV attributed to LiF in F1s spectra (Supplementary Fig. 9). The signature of LiF species was not seen in the postmortem XRD spectrum (to be discussed below) denoting its extremely low contribution/amorphous nature. Furthermore, *postmortem* SEM or TEM images of charged and discharged samples shown above did not demonstrate the formation of spherical agglomerates on the surface of CNFs often associated with LiF formation, further suggesting low LiF deposition originating from scattered sites^41^.

It is important to note that the carbonate electrolyte is expected to be stable and not decompose in the 0.8–4.2 V vs. Li/Li^+^ range and the formation of LiF can be attributed to the decomposition of the salt and not the organic electrolyte as no organic species can be seen in the cycled C1s and O1s spectra (Supplementary Fig. 10). Also, at lower scan rates, no current response was recorded corresponding to possible salt decomposition (Fig. 4b).

After the complete charge, S 2p spectra shows diminished Li_2_S peak at 161.8 eV and sulfur doublet peaks dominate the overall spectrum indicating reversible conversion of Li_2_S back to sulfur in the charge cycle. The presence of some Li_2_S even after charge may be associated with incomplete conversion due to the fast charge rate. The F1s spectra (Supplementary Fig. 9b) continue to show the presence of LiF and LiFxNy peaks from salt decomposition and salt species at 685.5 and 688 eV, respectively in charged samples.

Figure 7a–c shows the XRD patterns of the pristine, discharged, and charged γS-CNFs cathodes. The diffractogram of the pristine cathode as discussed earlier shows peaks of γ-S. During initial discharge, we observe a plateau at 2.0 V and the plateau continues to progress towards complete sulfur reduction at 1.0 V at a rate of 0.05 C. After complete reduction of cathodes, the diffraction pattern shows the presence of Li_2_S peaks (JCPDS 00-023-0369) at 2*θ* = 26.9, 31.2, 44.8, and 53.08 which correspond to reflections (111), (200), (220), and (311), respectively. It confirms the finding from XPS and TEM that the single plateau observed in the discharge cycle is associated with the reduction of γ-sulfur to lithium sulfide (Li_2_S). After the charge, interestingly, a completely different sulfur XRD pattern is observed. Such pattern has not been reported yet in the Li–S literature. These peaks are attributed to cyclo-deca-cyclo-hexa sulfur, also belonging to a monoclinic crystal structure family. No overlapping gamma monoclinic sulfur peaks were observed. This *post mortem* study demonstrates the complete conversion of γ-monoclinic sulfur to Li_2_S and back to a new sulfur monoclinic crystal phase. This is the first-ever study to report stability of such sulfur crystal structures in Li–S batteries and their operation in carbonate electrolytes. It has been previously reported in ether-based Li–S batteries that α-orthorhombic sulfur allotrope (the most stable sulfur allotrope at room temperature) indeed converts to the β-monoclinic phase and that phase dominates after the first charge cycle^39^. Using this analogy, we hypothesize that the monoclinic phase is thermodynamically more stable in the Li–S electrolyte medium and is therefore retained in our system even after the charging cycle. Nevertheless, we observe a unique monoclinic phase in these charged samples, which is different from the β-monoclinic phase seen in ether electrolyte, and this possibly plays a role in retaining single plateau behavior in charge-discharge profiles for over 4000 cycles. Further studies, particularly computational modeling and simulations, are necessary to understand the origin of this phenomenon.

## Conclusions

In this work, we synthesize and study a novel phase of sulfur (γ-monoclinic phase) in carbonate electrolyte-based lithium-sulfur batteries. Carbonate electrolytes, despite their tremendous commercial success in Li-ion batteries for the past three decades, are known to cause unfavorable and irreversible side reactions with intermediate sulfur reduction products (polysulfides) in Li–S batteries resulting in a complete cell shutdown. In our work, we demonstrate that despite an exposed “un-confined” deposition of the γ-monoclinic sulfur on the host carbon material, the carbonate-based battery exhibits high reversible capacity, which stabilizes to 800 mAh/g in the first few cycles and then it remains stable with a small 0.0375% decay rate over 4000 cycles. Fundamental electrochemical characterization and *post-mortem* XRD, XPS, SEM, and TEM studies on cycled cells reveal an altered redox mechanism that reversibly converts γ-monoclinic sulfur to Li_2_S without the formation of intermediate polysulfides eliminating irreversible side reactions for the entire range of 4000 cycles. Nevertheless, practical applications require far more aggressive optimizations with the large-scale continuous fabrication of CNFs, tuning its surface porosity, and finally additives in the electrolyte to stabilize the system to achieve commercial-grade performance. To the best of our knowledge, we are the first to report both the stabilization of γ-monoclinic sulfur at room temperature and its utilization in Li–S batteries. We believe, this work will trigger new fundamental research, especially to understand the sulfur phase-performance correlations in various electrolytes coupled with in situ/operando characterization to elucidate information on structure evolution, redox mechanisms, changes in the system environment contributing towards phase stability and ion transport properties. This will enable a deeper understanding of the system facilitating the commercialization of Li–S batteries.

## Methods

### Materials

Polyacrylonitrile (PAN, Mw 150 000 g mol^−1^), N, N-Dimethylformamide (DMF, purity 99.8%), Sulfur (S, purity 99.998% trace metals basis), ethylene carbonate (EC, purity ≥ 99%, acid < 10 ppm, H_2_O < 10 ppm), diethyl carbonate (DEC, purity ≥ 99%, acid < 10 ppm, H_2_O < 10 ppm),lithium nitrate (Sigma Aldrich) 1,2-dimethoxyethane (DME) (Sigma Aldrich), and lithium hexafluorophosphate (LiPF_6_, purity ≥ 99.99% trace metals basis, battery grade) were purchased from Sigma Aldrich. 1,3-dioxolane (DOL) (99.8%, anhydrous, stabilized with 75 ppm BHT) and lithium trifluoromethanesulfonate were purchased from Acros Organics. All chemicals were used without further processing.

### Material synthesis

#### Synthesis of CNFs

The free-standing CNFs were made by electrospinning^42^. Typically, 10 wt% polyacrylonitrile, was added to DMF and stirred overnight to form a polymeric solution. This solution was then loaded into a Becton Dickinson 5 mL syringe with a Luer lock tip and an 18-gauge stainless steel needle (Hamilton Corporation). The syringe with the needle was connected to a NE-400 model syringe pump (New Era Pump Systems, Inc.) to control the feeding rate of the solution. The grounded aluminum collector was placed 6 in. from the tip of the needle. Electrospinning was performed at room temperature with a relative humidity below 15%. A potential difference of 7–8 KV (Series ES -30 KV, Gamma High Voltage Research, Inc.) was applied between the collector and the tip of the needle. The flow rate of the solution was kept constant at 0.2 mL h^−1^. The as-spun nanofibers were collected and stabilized in a convection oven at 280 °C for 6 h in air atmosphere. The stabilized nanofiber mats were then placed in alumina plates and carbonized in a nitrogen environment up till 900 °C at a ramp rate of 2.5 °C min^−1^ and then activated under CO_2_ flow for 1 h in a horizontal tube furnace (MTI. Corp). The furnace was then cooled at 2 °C min^−1^ until it reached room temperature.

#### Monoclinic γ-sulfur deposition on CNFs

The free-standing CNF mats were punched with stainless steel die (*ϕ* = 11 mm) and dried at 150 °C overnight under vacuum. The CNF discs were then weighed and placed in an in-house developed autoclave (Stainless steel 316) and subjected to 180 °C for 24 h in an oven. The autoclave consisted of a sulfur reservoir at the bottom and a perforated disk for placing electrodes at the top. After 24 h the autoclave was cooled to room temperature slowly in a span of 6–8 h. The electrodes were weighed and transferred in an Argon-filled glove box via overnight room temperature vacuum drying in the antechamber for battery fabrication.

### Characterization

#### Material characterization

Morphological and elemental characterization of the nanofibers was conducted using an SEM (Zeiss Supra 50 VP, Germany) equipped with energy-dispersive X-ray spectroscopy with an in-lens detector and 30 µm aperture. XRD patterns were acquired on a diffractometer (Rigaku Smartlab, Tokyo, Japan) using Cu K_α_ radiation (40 kV and 44 mA) with a step size of 0.02° in the 2*θ* range of 10°–70°. The surface chemistry of the samples was analyzed using XPS spectra (Physical Electronics Versa Probe 5000 spectrometer with monochromatic Al Kα as an excitation source) with a spot size of 200 μm and pass energy of 23.5 eV. A step size of 0.5 eV was used to gather the high-resolution spectra. CasaXPS Version 2.3.19PR1.0 software was used for spectra analysis. The XPS spectra were calibrated by setting the valence edge to zero, which was calculated by fitting the valence edge with a step-down function and setting the intersection to 0 eV. The background was determined using the Shirley algorithm, which is a built-in function in the CasaXPS software. TGA data were collected on TA Instruments 2950 (TA Instruments, New Castle, DE) under steady argon flow at a heating ramp rate of 5 °C min^−^^1^. Nitrogen adsorption–desorption analysis of the freestanding nanofiber mats was performed at −196.15 °C on an automated gas sorption analyzer (AutoSorb iQ2, Quantachrome Instruments). The sample was degassed overnight at 150 °C under N_2_ flow prior to this analysis.

#### Electrochemical characterization

Electrochemical measurements were conducted by assembling 2032—type coin cells (MTI and Xiamen TMAX battery equipment) in an argon-filled glove box (MBraun Labstar pro, MB 10 G, H_2_O, and O_2_ < 1 ppm). As-transferred electrodes were used as working electrodes and 13 mm lithium discs punched from Lithium foil (Alfa Aesar, 0.75 mm thick) were used as counter/reference electrodes. To improve the mass loading, cathodes were stacked onto each other. A typical sulfur weight loading of around 45–50% was used with a mass loading of around 0.5–5 mg cm^−2^ for electrochemical testing. The ether electrolyte was prepared by dissolving 1.0 M Lithium bis(trifluoromethanesulfonyl)imide in a solvent mixture of DME: DOL 1:1 volume ratio with 1 wt% LiNO_3_. The carbonate electrolyte consisted of 1 M LiPF_6_ in 1:1 volume ratio of EC: DEC. The E/S ratio was kept constant at 20 for all electrochemical testing. A tri-layer membrane 25 μm thick (2325, Celgard Inc) was used as a separator. The galvanostatic charge-discharge measurements were carried out in a potential range of 1.0–3.0 V vs. Li/Li^+^ using Maccor 4000 and Neware BTS 4000 battery cyclers. The CV measurements were performed in a potential range of 1.0–3.0 V vs. Li/Li^+^ at a range of scan rates from 0.01 to 0.5 mV·s^−1^ using a multi-channel potentiostat (Biologic VMP3). The capacity calculations were done considering the weight of sulfur in the cathodes and the C rates were calculated based on 1 C = 1675 mA·g^−1^. The long cycling tests were done at lower loading of 0.5 mg·cm^−2^ and high loading cells were tested at 5 mg·cm^−2^ with the same E/S ratio of 20. EIS measurements were performed between 100 and 100 MHz frequency range using an AC perturbation of 10 mV RMS amplitude (Biologic VMP3).

## Supplementary information

Supplementary file

## Data Availability

The data that support the findings of this study are available from the corresponding author on reasonable request.
