# Supplementary material for: Stabilization of gamma sulfur at room temperature to enable the use of carbonate electrolyte in Li-S batteries
Source: Commun Chem. 2022 Feb 10;5:17. doi: 10.1038/s42004-022-00626-2 (PMC9814344; doi:10.1038/s42004-022-00626-2)
Supplement: Supplementary file 1 — Supplementary file [file 42004_2022_626_MOESM1_ESM.pdf]

Supplementary Information

Stabilization of Gamma Sulfur at Room Temperature to Enable the Use of Carbonate Electrolyte in Li-S batteries

Rahul Pai[†], Arvinder Singh[†], Maureen H. Tang[†], Vibha Kalra[†]*

[†]Department of Chemical and Biological Engineering, Drexel University,

3141 Chestnut Street, Philadelphia, PA-19104

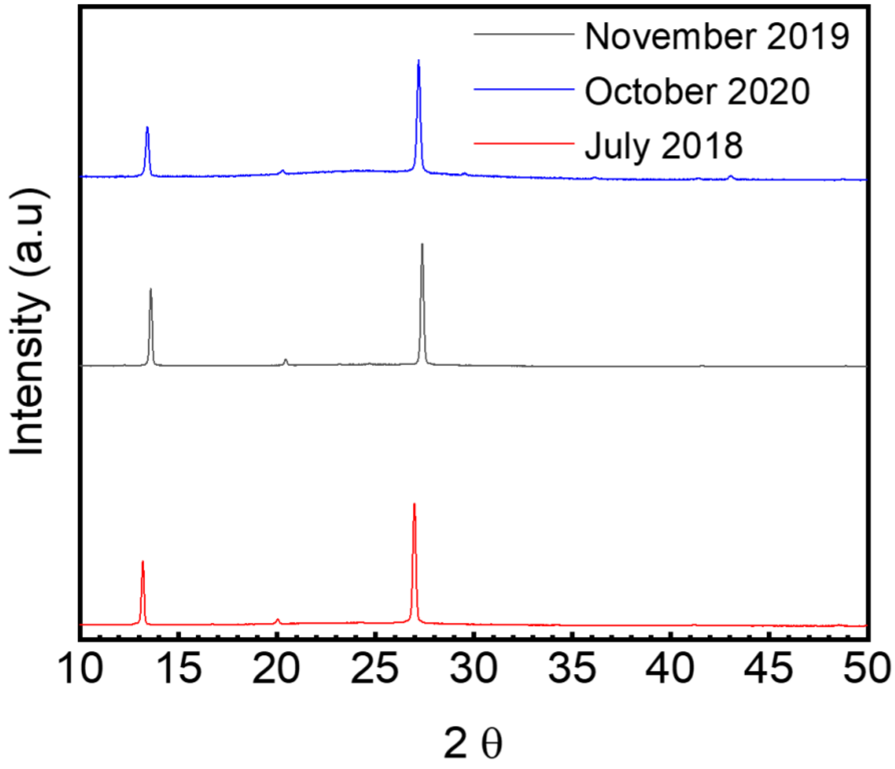

Supplementary figure 1: XRD pattern for stability of γS-CNFs stored over 2 years in glove box

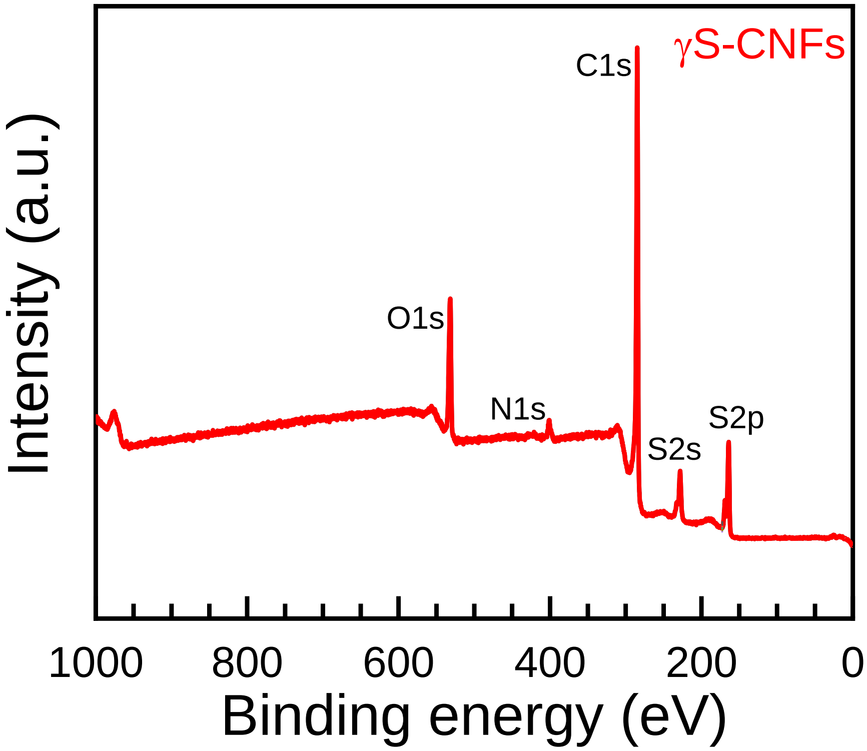

Supplementary figure 2: Survey XPS spectra of pristine γS-CNFs

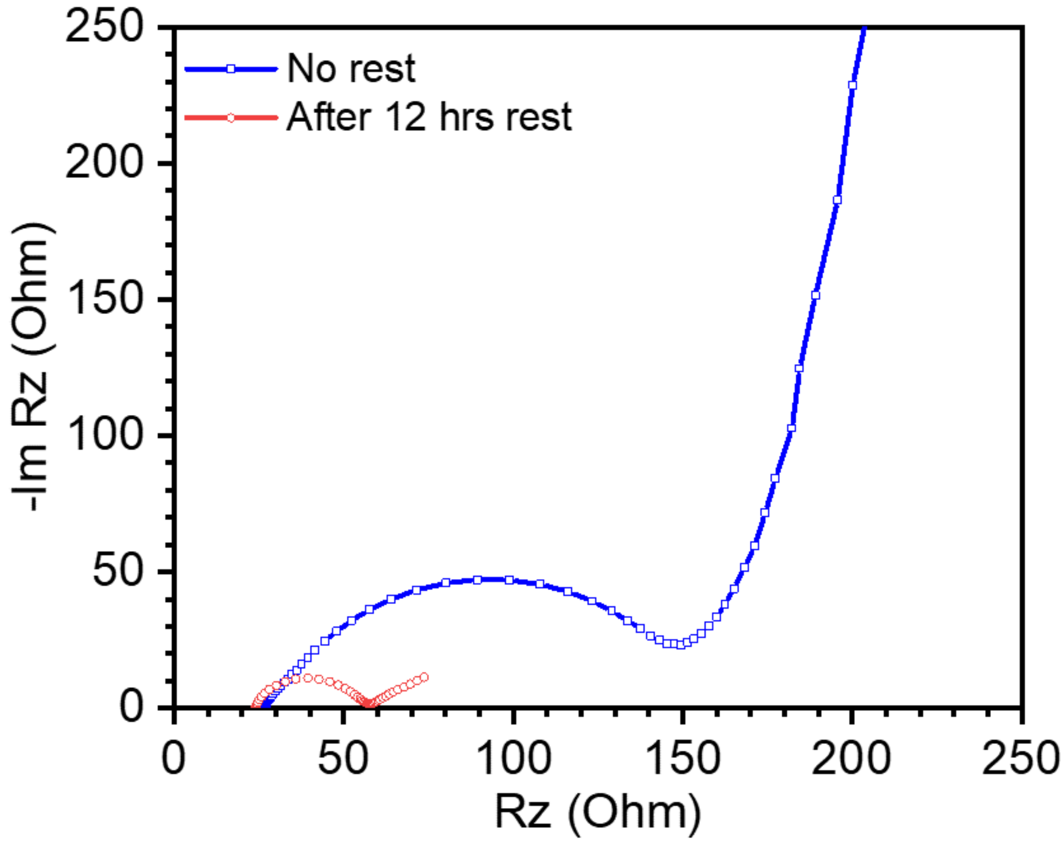

Supplementary figure 3: Nyquist plot of γS-CNFs in EC:DEC electrolyte without and with 12 hours rest time.

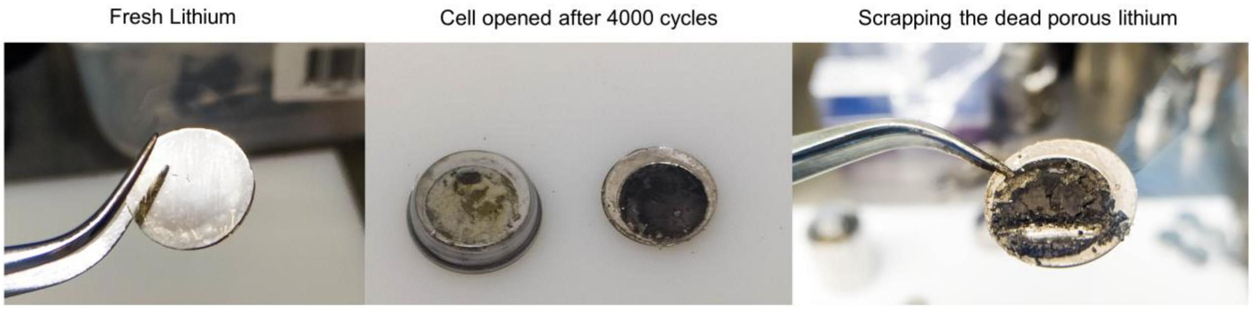

Supplementary figure 4: Digital images of fresh lithium and after cycling for 4000 cycles.

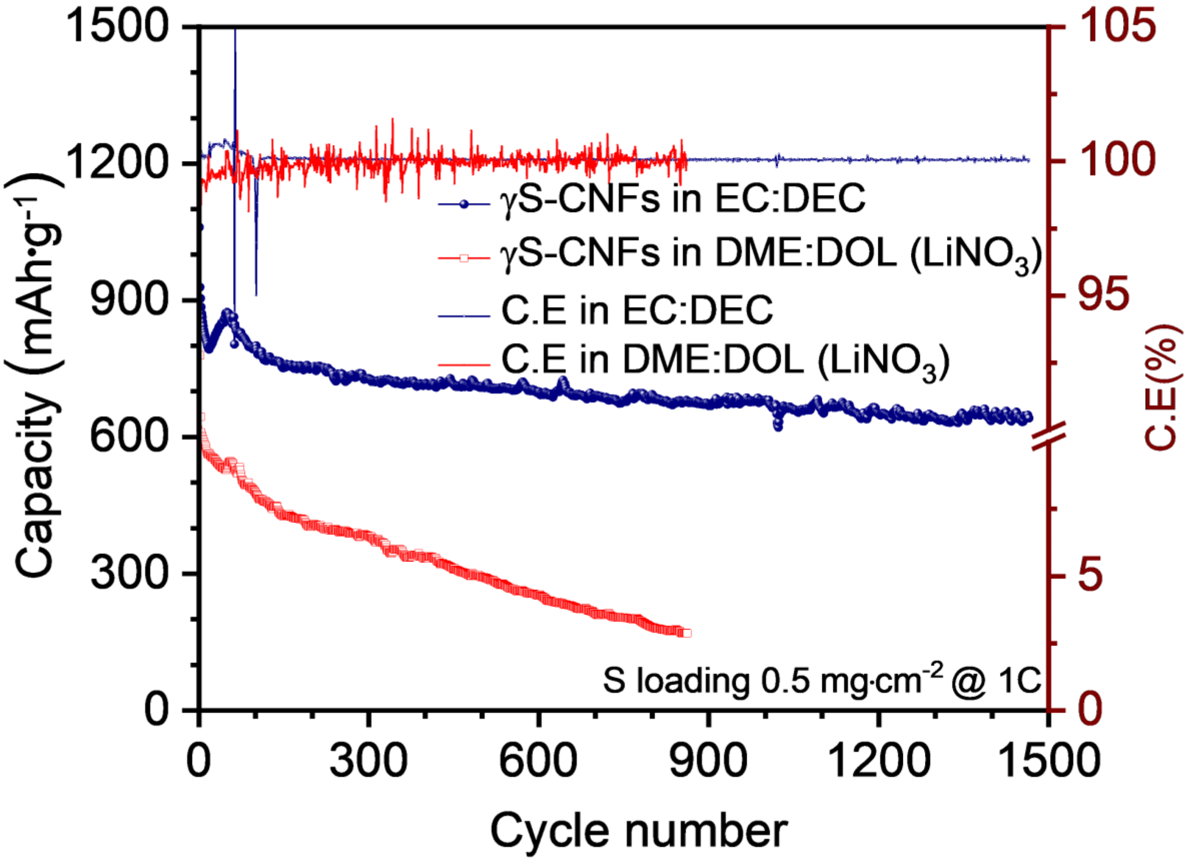

Supplementary figure 5: Performance comparison of γS-CNFs in carbonate (1M $LiPF_6$ in EC:DEC) and ether (1 M LiTFSi in DME:DOL) electrolyte. Sulfur loading of 0.5 mg/cm^2 and E/S ratio of 20.

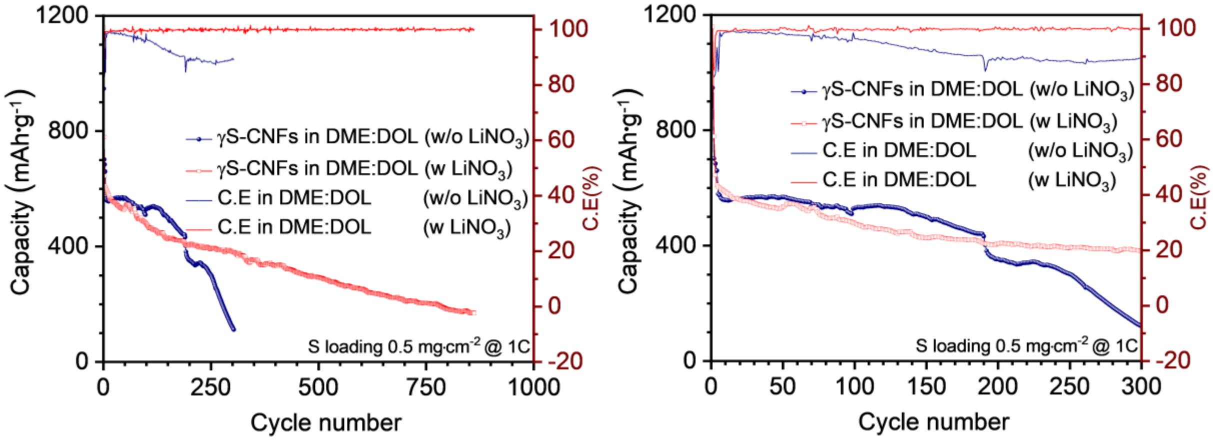

Supplementary figure 6: Capacity and CE comparison of γS-CNFs in ether electrolyte with and without $LiNO_3$. Zoomed image is on the right. Sulfur loading of 0.5 mg/cm^2 and E/S ratio of 20.

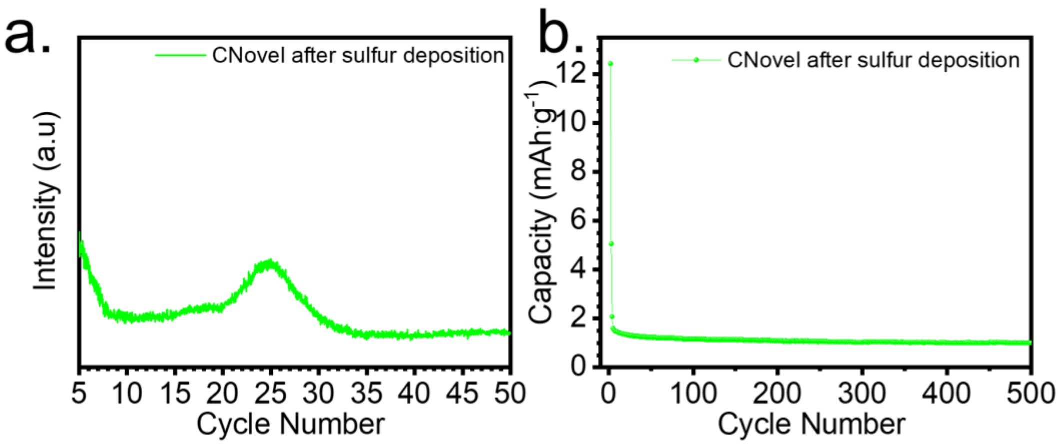

Supplementary figure 7: a. XRD pattern of CNovel after sulfur deposition process, b. Capacity as a function of cycle life with CNovel after sulfur deposition as host material.

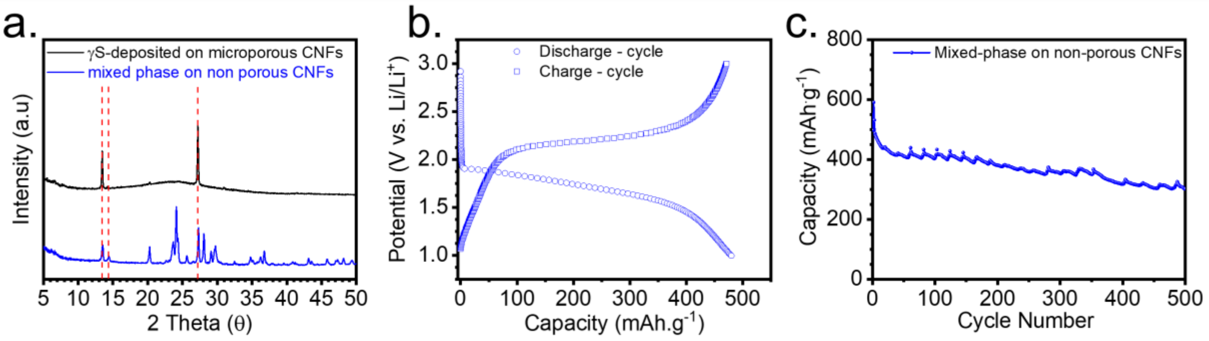

Supplementary figure 8: a. XRD pattern of gamma-sulfur deposited on carbon nanofibers and the mixed-phase from scrapped powder from deposition assembly mixed with non-porous CNFs, b. Charge-discharge profile utilizing mixed-phase powder in the 5th cycle in carbonate electrolyte (1M $LiPF_6$ in EC:DEC), c. Cycling performance as utilizing mixed-phase powder. Sulfur loading of 1 mg/cm^2 and E/S ratio of 20.

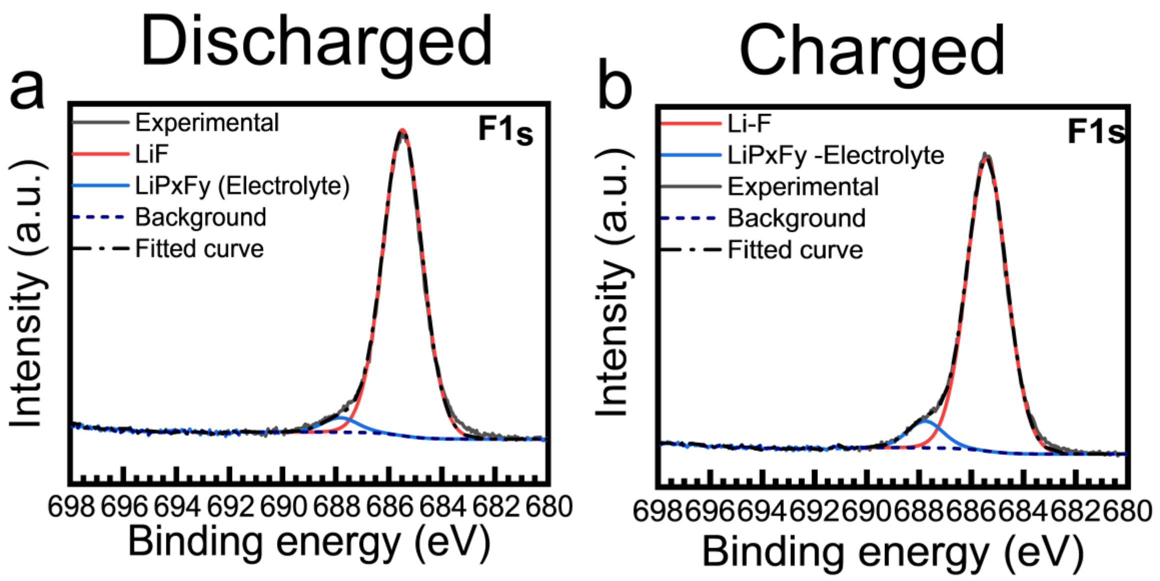

Supplementary figure 9: XPS spectra of F1s region of charged and discharged samples

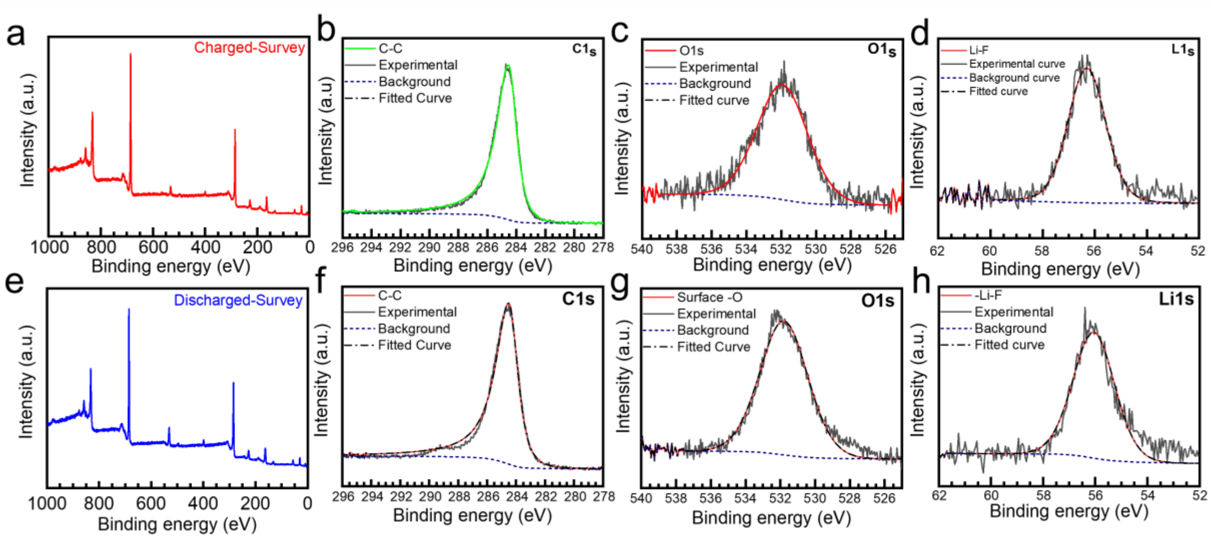

Supplementary figure 10: XPS spectra of charged samples (a,b,c,d) and discharged samples (e,f,g,h)

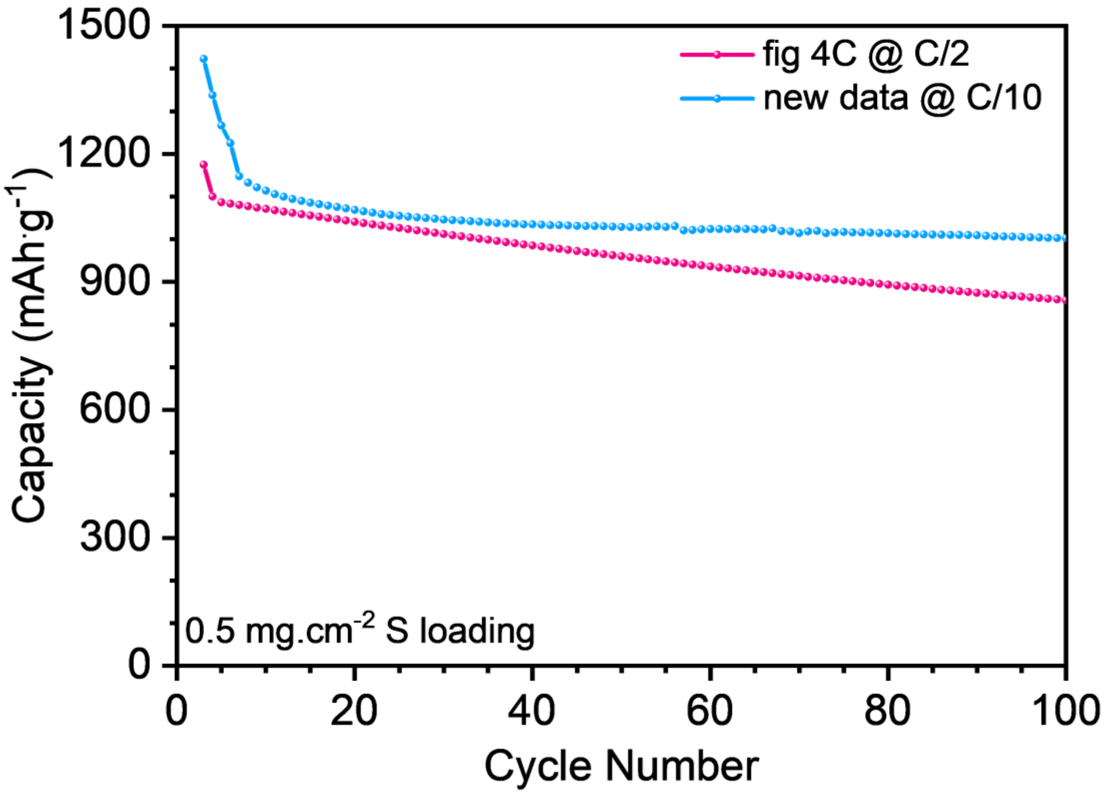

Supplementary figure 11: Cell comparison for high and low C (0.1 C and 0.5 C) rate at same sulfur loading. Sulfur loading of 0.5 mg/cm^2 and E/S ratio of 20.

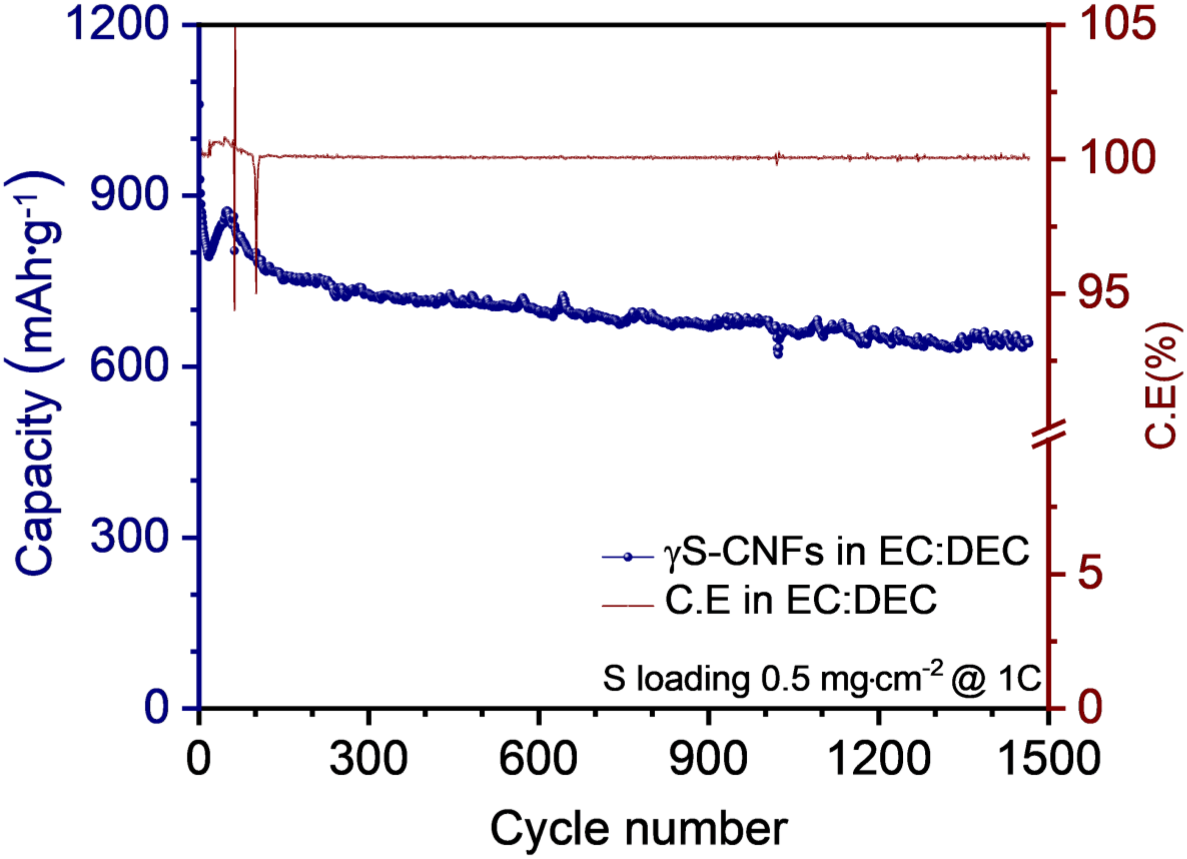

Supplementary figure 12: Cycling performance of γS-CNFs in carbonate electrolyte at 1C. Sulfur loading of 0.5 mg/cm^2 and E/S ratio of 20.
